# Death of Dr. Bratton

**Published:** 1897-11

**Authors:** 


### DEATH OF DR. BRATTON.

A SINGULAR FATALITY.—The JOURNAL's readers will recall our account of the usurpation of authority at Sabine Pass by the Marine Hospital Service, Surgeon Magruder, of that service, displacing the State's true and tried quarantine officer, Dr. A. N. Perkins. The sequel was a sad one, and looks like fate. The Marine Hospital Service recalled Dr. Magruder, and sent in his stead Passed Assistant Surgeon Bratton.

It looks to us to have been a very injudicious selection to send to such a place an assistant surgeon, who, as he confessed to Dr. Perkins, knew nothing of quarantine, and who had never seen yellow fever.

Dr. Perkins writes to the State Health Officer that Dr. Bratton was a pleasant and agreeable young man, and that he was much pleased with the manner in which the State quarantine men disinfected a ship. He had witnessed the process of disinfecting a large vessel, and intended next day to report to Surgeon General Wyman that his presence at Sabine Pass was unnecessary, and that the quarantine was as efficiently administered by the State as was possible. He said he would "go and take another look at the vessel." It was late in the afternoon, and his launch was waiting to take him back to his quarters. Night came, and he did not return. The boatmen became alarmed at his absence, informed Dr. Perkins, and together they instituted a search for him. He was found lying in the hold of the ship in a state of total unconsciousness, from which he never rallied; but died within eight or ten hours. It seems that as it was dark in the hold, he made a misstep, and fell about fifteen feet, striking the back of his head on a cast iron knee. There was no fracture of the skull.

The JOURNAL is pained to have to record so tragic an ending to the controversy between the State and national quarantine departments.

Surgeon General Wyman, of the Marine Hospital Service, has issued a circular letter to the officers of his department, announcing the death of Dr. Bratton, and paying a high tribute to his worth and ability. We extract from Dr. Wyman's letter the following:

Dr. Wm. DuBose Bratton was born in South Carolina, June 23, 1860. He graduated at the Medical College of South Caroline March 1, 1884; entered the Marine Hospital Service April, 1885, as assistant surgeon, and assigned to duty at New York; served as Medical Officer of the revenue cutter Corwin, and later of the revenue cutter Bear, in Alaskan waters. April, 1888, he was made Passed Assistant Surgeon, and was in command of the Marine Hospital Service at Portland, Oregon; later, in command of the service at Buffalo, N. Y. He was the victim of pulmonary tuberculosis, and was placed on waiting orders in Arizona, and later in Alburquerque, N. M., where he hoped to obtain the benefit of the dry climate. After a two years' residence there, he reported March, 1897, his gradual return to health, and asked for a restoration to active service. He was advised to wait awhile longer, but in August, 1887, he was sent to Sabine Pass to relieve Dr. Magruder, as we have seen.

